# MUC1 attenuates neutrophilic airway inflammation in asthma by reducing NLRP3 inflammasome-mediated pyroptosis through the inhibition of the TLR4/MyD88/NF-κB pathway

**DOI:** 10.1186/s12931-023-02550-y

**Published:** 2023-10-25

**Authors:** Lu Liu, Ling Zhou, Lingling Wang, Zhenyu Mao, Pengdou Zheng, Fengqin Zhang, Huojun Zhang, Huiguo Liu

**Affiliations:** 1 Department of Respiratory and Critical Care Medicine, Key Laboratory of Pulmonary Diseases of Health Ministry, Tongji Hospital, Tongji Medical College, Huazhong University of Science and Technology, Wuhan, China; 2 Department of Respiratory Medicine, The First People’s Hospital of Yunnan Province, Kunming, Yunnan China; 3 The Affiliated Hospital of Kunming University of Science and Technology, Kunming, Yunnan China; 4 Department of Respiratory and Critical Care Medicine, Renmin Hospital of Wuhan University, Wuhan, China

**Keywords:** Asthma, MUC1, Pyroptosis, Inflammation

## Abstract

**Background:**

Neutrophilic airway inflammation is a challenge in asthma management and is associated with poor patient prognosis. Mucin 1 (MUC1), which contains a cytoplasmic tail (MUC1-CT), has been found to mediate glucocorticoid sensitivity in asthma; however, its role in modulating neutrophilic airway inflammation in asthma remains unknown.

**Methods:**

Human-induced sputum cells were collected from healthy participants (n = 12), patients with mild-to-moderate asthma (n = 34), and those with severe asthma (n = 18). In vitro human lung bronchial 1 epithelial cell line (BEAS-2B) was transfected with small interfering RNA against MUC1 (MUC1-siRNA) and then stimulated by lipopolysaccharide (LPS), where some cells were pretreated with a TLR4 inhibitor (TAK-242). In vivo mouse model of asthmatic neutrophil airway inflammation was induced by ovalbumin (OVA)/LPS. Some groups were intraperitoneally injected with MUC1-CT inhibitor (GO-203) and/or TAK-242 .

**Results:**

The mRNA expression of MUC1 was downregulated in the induced sputum of patients with asthma and correlated with asthmatic neutrophilic airway inflammation. The mRNA expressions of TLR4, MyD88, nucleotide-binding oligomerization domain-like pyrin domain-containing protein 3 (NLRP3), caspase-1, interleukin (IL)-18, and IL-1β in induced sputum cells of patients with asthma were upregulated and related to the mRNA expression of MUC1. LPS activated the TLR4 pathway and NLRP3-mediated pyroptosis in BEAS-2B cells in vitro, which were significantly aggravated after MUC1-siRNA transfection. Furthermore, MUCl-CT interacted with TLR4, and the interaction between TLR4 and MyD88 was significantly increased after MUCl-siRNA transfection. Moreover, TAK-242 ameliorated TLR4/MyD88/nuclear factor kappa B (NF-κB) pathway activation, NLRP3 inflammasome-mediated pyroptosis, and neutrophilic inflammation exacerbated by MUC1 downregulation. GO-203 exacerbated TLR4/MyD88/NF-κB pathway activation in vivo, and NLRP3 inflammasome-mediated pyroptosis reduced in a mouse model of asthmatic neutrophil airway inflammation induced by OVA/LPS; these pathological changes were partially alleviated after TAK-242 application.

**Conclusion:**

This study revealed that MUC1 downregulation plays an important role in asthmatic neutrophilic airway inflammation. MUC1-CT reduces NLRP3 inflammasome-mediated pyroptosis by inhibiting the activation of the TLR4/MyD88/NF-κB pathway, thereby attenuating neutrophil airway inflammation in patients with asthma.

**Supplementary Information:**

The online version contains supplementary material available at 10.1186/s12931-023-02550-y.

## Background

Asthma is a heterogeneous chronic respiratory inflammatory disease characterized by reversible airflow limitation, mucus hypersecretion, and airway hyperresponsiveness. The incidence rate of asthma in different countries ranges from 1 to 18%. Asthma can be classified into different inflammatory endotypes based on the proportion of inflammatory cells in the induced sputum [1, 2]. Neutrophilic asthma accounts for approximately 20–30% of all adult asthma cases [3]. Moreover, patients with asthma having neutrophilic airway inflammation frequently present with more late-onset, severe, or steroid-resistant asthma and are difficult to treat [4, 5]. Thus, the possible mechanism and effective therapeutic targets for neutrophilic airway inflammation should be explored in patients with asthma.

Pyroptosis is a proinflammatory programmed cell death characterized by cell lysis and proinflammatory cytokine release [6]. Notably, nucleotide-binding oligomerization domain-like pyrin domain-containing protein 3 (NLRP3) inflammasome-mediated caspase-1 activation is the canonical pathway of pyroptosis [7, 8]. Increasing evidence has demonstrated the important role of NLRP3 inflammasome-mediated pyroptosis in asthmatic neutrophilic airway inflammation. Simpson et al. [9] revealed significantly elevated gene expression of NLRP3, caspase-1, and interleukin (IL)-1β in induced sputum of patients with neutrophilic asthma. Richard Y Kim et al. [10] proposed that neutrophilic airway inflammation, disease severity, and steroid resistance in asthma were correlated with NLRP3 and IL-1β expression. Furthermore, inhibition of NLRP3-mediated pyroptosis has been reported to ameliorate airway neutrophilic inflammation in a murine asthma model [11, 12].

Mucin 1 (MUC1), a transmembrane glycoprotein in the mucin family, primarily consists of two noncovalently bound subunits, i.e., an N-terminal extracellular subunit and a C‐terminal subunit containing a cytoplasmic tail (MUC1‐CT). Unlike most other membrane-bound mucins, MUC1 is not only expressed in various epithelial cells but also in immune cells, such as neutrophils and macrophages [10]. Moreover, MUC1-CT can be shed from the cell membrane, interact with cell membrane receptors, or undergo nuclear translocation to regulate cell signal transduction [10, 13]. In recent years, several studies have revealed that MUC1 has a potent anti-inflammatory function in the respiratory system [13, 14], and its role in asthma has also received some attention. Javier Milara et al. [15] revealed downregulated MUC1 expression in bronchial epithelial cells and peripheral blood neutrophils of patients with severe asthma, which was inversely correlated with daily doses of inhaled corticosteroids. Based on the theory that correct MUC1-CT/GRα complex formation and nuclear translocation are important to enhance corticosteroid anti-inflammatory inducible genes, they proposed that MUC1 deficiency impairs corticosteroid insensitivity in patients with asthma [16]. Some studies have revealed that MUC1 can regulate some programmed cell death pathways, such as apoptosis [17], necroptosis [18, 19], and ferroptosis [20]. However, the influence of MUC1 on NLRP3-mediated pyroptosis remains unknown.

Toll-like receptor 4 (TLR4), which is the earliest discovered pattern recognition receptor in the TLR family, plays an important role in immune system function and inflammatory diseases [21]. Studies have revealed that TLR4/MyD88/NF-κB pathway suppression can attenuate pathological mechanisms of asthma [22, 23]. Previous studies have reported that TLR4 is the upstream signal of NLRP3 inflammasome activation, and TLR4 can interact with its downstream partner myeloid differentiation primary response 88 (MyD88) on the cell membrane and activate nuclear factor kappa-light-chain enhancer of activated B cells (NF-κB) to stimulate pyroptosis in various cell types [14, 24, 25]. Furthermore, studies have revealed that MUC1 is an upstream regulator of the TLR4/MyD88/NF-κB signaling pathway [26, 27].

This study aimed to explore the influence of MUC1 on neutrophil airway inflammation in patients with asthma and verify the underlying mechanism associated with the TLR4/MyD88/NF-κB pathway and NLRP3 inflammasome-mediated pyroptosis.

## Methods

### Study subjects

Adult patients with physician-diagnosed asthma were recruited if they met the following inclusion criteria: recurrent episodes of wheezing, dyspnea, cough, and sputum production; either methacholine PD20 of < 2.505 mg or bronchodilation FEV1 change of > 200 mL; and 12% successfully induced sputum. The exclusion criteria were as follows: smokers, including those who quit smoking for < 6 months; those with an acute exacerbation in the last 1 month; those with respiratory infection in the last 2 weeks; those comorbid with chronic obstructive pulmonary disease, bronchiectasis, or interstitial pneumonia; those who were pregnant or breastfeeding; and those recently diagnosed with lung cancer or other solid organ malignancy. Asthma severity was retrospectively assessed based on the treatment level required to control symptoms at least 2–3 months according to the Global Initiative for Asthma 2021 [28]. Severe asthma (SA) is asthma which requires optimized treatment with high-dose ICS/LABA to prevent it from becoming uncontrolled or which remains uncontrolled despite such treatment. Healthy controls (HC) had no history of smoking for the last 6 months; no family history of asthma; no history of chronic respiratory disease and respiratory infection in the last 2 weeks; and no severe systemic disease. The induced sputum cells and supernatant of the participants were stored at − 80 °C for subsequent experiments.

All participants provided informed consent. The Ethics Committee of Tongji Hospital, Tongji Medical College, Huazhong University of Science and Technology approved the study.

### Cell culture

Human bronchial epithelial cells (BEAS-2B, ATCC; Manassas, VA, USA) were cultured in Dulbecco’s Modified Eagle Medium (Gibco, USA) supplemented with 10% fetal bovine serum (Gibco, USA) in a 5% CO_2_ atmosphere at 37 °C. Cells were stimulated with 5 µg/mL of lipopolysaccharide (LPS) (Cat #: L2880, Sigma-Aldrich, USA) and 100 nM of TAK-242 (Cat #: HY-11,109, MedChemExpress, NJ, USA), and TAK-242 was preadded to the medium 2 h before LPS.

### Lactate dehydrogenase (LDH) release assay

Cells were inoculated in 96-well plates at a density of 5 × 10^3^ cells/well, and the culture supernatants were collected after drug treatment according to experimental requirements. Then, the LDH Cytotoxicity Assay Kit (Beyotime, Shanghai, China) was used to quantify LDH release in the cell culture supernatant following the manufacturer’s instructions. Absorbance at 490 nm was measured using a microplate reader.

### Small interfering RNA (siRNA) transfection

The transfection reagent lipofectamine 3000 (Invitrogen, Carlsbad, CA, USA) was used to transfect BEAS-2B cells with human MUC1-siRNAs or NC-siRNA (Guangzhou, China) for 24 h. The MUC1-siRNA sequence was 5′-GTTCAGTGCCCAGCTCTAC-3′.

### Animal model and treatments

Female C57BL/6J mice (6–8 weeks old) were obtained from Gempharmatech (Jiangsu, China). All animals were maintained in a specific pathogen-free facility with free access to standard fodder and water and kept in a controlled environment (22 °C ± 2 °C, 55% ± 5% humidity) under a 12-h light/dark cycle. The animal protocols were approved by the Animal Care and Use Committee at the Tongji Hospital (Certificate number: TJH-202,209,007, Approval date: September 2022 to November 2022).

Mice were divided into four groups (n = 6/group): control, OVA/LPS, OVA/LPS + GO-203, and OVA/LPS + GO-203 + TAK-242. Mice in the OVA/LPS, OVA/LPS + GO-203, and OVA/LPS + GO-203 + TAK-242 groups were sensitized by intraperitoneally injecting 100 µg of sensitized grade V OVA (Sigma-Aldrich, USA) and 1 mg of aluminum hydroxide (Thermo Fisher Scientific, USA) on days 0, 7, and 14. Subsequently, the sensitized animals were intranasally administered with 75 µg of OVA and 3.5 µg of LPS on days 21, 22, and 23. Mice in the control group were intraperitoneally injected and intranasally administered with an equal volume of phosphate-buffered saline (PBS) on the corresponding days. Mice in the OVA/LPS + GO-203 and OVA/LPS + GO-203 + TAK-242 groups were intraperitoneally injected with the MUC1 inhibitor GO-203 (MedChemExpress, NJ, USA) (20 mg/kg) 2 h before each challenge. Mice in the OVA/LPS and OVA/LPS + GO-203 groups were intraperitoneally injected with PBS. Mice in the OVA/LPS + GO-203 + TAK-242 group were intraperitoneally injected with the TLR4 inhibitor TAK-242 (MedChemExpress, NJ, USA) (3 mg/kg) 2 h before the challenge. The mice in the other groups were intraperitoneally injected with the same volume and concentration of DMSO solvent. All mice were anesthetized, and bronchoalveolar lavage fluid (BALF) and lung tissue were collected for subsequent experiments on day 24.

### Hematoxylin and eosin (H&E) staining and periodic acid–Schiff (PAS) staining

The lung tissues were fixed in 4% paraformaldehyde for 24 h. Then, the sections were embedded in paraffin, cut into 4-µm-thick sections, and subjected to H&E staining. The scoring criteria for peribronchial inflammation were as follows: 0, no inflammatory cells; 1, a small number of cells; 2, one layer of cells; 3, 2–4 layers of cells; and 4, > 4 layers of cells around the bronchi. The lung tissue sections were subjected to PAS staining to determine the abundance of PAS-positive cells in the airway and scored as follows: 0, the proportion of the positive cells of < 5%; 1, 5–25%; 2, 25–50%; 3, 50–75%; and 4, > 75%.

### Reverse transcription-quantitative polymerase chain reaction (RT-qPCR)

Trizol reagent (Takara Co., Dalian, China) was used to extract the total RNA from induced sputum cells, BEAS-2B cells, or lung tissues, and PrimeScript RT reagent Kit (Takara) was used for reverse transcription in the first-strand cDNA synthesis reaction. An SYBR Premix Ex Taq (Takara) was used for PCR reaction on Bio-Rad C1000 Thermal Cycler. The relative mRNA expression was analyzed using the 2^−ΔΔCt^ method, and the expression level was presented as fold change relative to the control. Primer sequences were synthesized by Sangon Biotech (Shanghai, China) (Table S1).

### Immunoblot and coimmunoprecipitation (CO-IP) analyses

Total protein was extracted from cultured cells and lung tissues using RIPA lysis buffer (Aspen Biological, Wuhan, China). The Nuclear and Cytoplasmic Protein Extraction Kit (Boster Biotech, Wuhan, China) was used to extract cytoplasmic and nuclear protein. BCA (Aspen) was used to measure protein concentration. The protein samples were separated via 12.5 SDS-PAGE (Yeasen Biotechnology, Shanghai, China) and then transferred onto polyvinylidene fluoride membranes (Roche, UK). Subsequently, the membranes were blocked with 5% nonfat dried milk in Tris-buffered saline with Tween 20 (TBST) for 90 min at 25 °C. Then, the membranes were incubated in the corresponding primary antibody overnight at 4 °C on a shaker. Subsequently, the membranes were washed using TBST and incubated with an appropriate HRP-labeled secondary antibody for 90 min at room temperature on a shaker. Thereafter, a ChemiDoc XRS gel imaging system (Bio-Rad, Hercules, CA, USA) was used to assay the membranes that were washed again with TBST. Image J (NIH Image, Bethesda, MD) was used to quantitatively analyze the protein bands. GAPDH was included as a reference. Table S2 shows the primary antibodies used for western blotting.

CO-IP proteins were extracted from cultured cells using NP40 lysis buffer (#B1027,Wuhan Promoter Biological CO., LTD). Equal amounts of protein aliquots of cell lysates were incubated overnight at 4 °C with immunoprecipitating antibody against MUC1-CT, MyD88, TLR4, or rabbit IgG (Abmart, B30011). Subsequently, protein A/G agarose beads (Abmart, Shanghai, China; A10001) were added and incubated together for 2 h. Then, the beads were washed with lysis buffer and eluted by boiling in loading buffer. Immunoprecipitated proteins were subjected to immunoblot analysis for the protein of interest. HRP-conjugated goat anti-rabbit IgG heavy chain (Abclone, AS063) was used as the secondary antibody for CO-IP.

### Immunofluorescence staining

BEAS-2B cell climbing films and lung tissue paraffin sections were immunostained according to the instructions of the Universal IF Toolkit (Abkine, Wuhan, China). In brief, BEAS-2B cell climbing films were washed twice with PBS, fixed in 4% paraformaldehyde at room temperature for 30 min, and rinsed with PBS. Afterward, the cell slides were incubated with immunostaining permeabilization buffer at room temperature for 40 min. The paraffin section was dewaxed in xylene and ethanol with gradient concentration, and then the antigen was repaired with EDTA antigen retrieval solution at a pH of 8.0 in microwave oven and washed with PBS. Subsequently, the cell slides or lung tissue sections were incubated with goat serum blocking buffer for 40 min at room temperature and with corresponding primary antibodies overnight at 4 °C. The next day, the cell slides or tissue sections were washed with antibody wash buffer and incubated with the corresponding fluorescent secondary antibody (Dylight 488 or A594, Goat Anti-Rabbit-IgG) for 1 h in dark. Then, they were re-stained with DAPI for 15 min and sealed with superKine™ snhanced antifade mounting medium. The slides or sections were visualized at ×400 under a microscope.

### Statistical analysis

Data analysis was performed using the GraphPad Prism 9 software (GraphPad, San Diego, California). Normally distributed data are presented as mean ± standard deviation (SD), and student’s t-test or one-way analysis of variance was used to compare these data among the groups. Non-normally distributed data are presented as median (Q1, Q3); Kruskal–Wallis analysis was used to compare these data among the groups, and Spearman’s rank order correlation was used for correlation analysis. Two-sided *p*-values of < 0.05 were considered statistically significant and expressed as * *p* < 0.05; ** *p* < 0.01; *** *p* < 0.001; and **** *p* < 0.0001.

## Results

### Subject characteristics

First, we collected induced sputum samples from 64 patients to detect whether MUC1 expression is altered in the airways of patients with asthma. Table 1 summarizes the patient characteristics. No significant differences were found in age, sex, and body mass index among the healthy (n = 12), mild-to-moderate asthma (MMA) (n = 34), and SA (n = 18) groups. The forced vital capacity in the first second (FEV1) of the SA group was significantly lower than that of the MMA group. The predicted FEV_1_% of the SA group was significantly lower than that of the healthy and MMA groups. The forced vital capacity in the first second/forced vital capacity (FEV_1_/FVC) of the SA group was significantly lower than that of the healthy group. The proportion of neutrophils in the induced sputum of the MMA and SA groups was higher than that in the induced sputum of the healthy group, and the proportion of SA was significantly higher than that of MMA.

**Table 1:** Subjects characteristics

	HC(n = 12)	MMA(n = 34)	SA(n = 18)	Overall *P*
Age (y)	44.92 ± 11.38	38.94 ± 12.30	45.83 ± 12.89	0.8385
Male n. (%)	7(58.33)	14(41.17)	6(33.33)	0.3915
BMI (kg/m^2^)	24.40(21.83, 27.93)	22.85(21.58, 25.83)	23.70(20.48, 26.85)	0.7185
FEV_1_ (L)	2.96 ± 0.66	2.98 ± 0.72	2.50 ± 0.62	0.0528
FEV_1_(%predicted)	100.35 ± 17.15	97.62 ± 12.90	85.71 ± 9.48 ^§K^	0.0097
FEV_1_/FVC (%)	78.33 ± 4.40	74.59 ± 7.31	70.15 ± 6.60 ^§^	0.0056
Induced sputum characteristics				
Neutrophils (%)	35.90(28.00, 40.45)	56.40(50.90, 63.68) ^§^	67.90(58.65,70.00) ^§K^	< 0.0001
Eosinophils (%)	0.30(0.03, 0.65)	5.75(1.80, 7.55) ^§^	9.15(5.22, 13.65) ^§K^	< 0.0001
Macrophages (%)	58.70(52.90, 67.78)	31.90(26.85, 36.00) ^§^	19.65(15.88, 22.68) ^§K^	< 0.0001
Lymphocytes (%)	5.75(5.03, 6.18)	5.65(5.08, 6.63)	6.05(5.28, 6.80)	0.3569

HC = Healthy controls (n = 12), MMA = mild-to-moderate asthma (n = 34), SA = severe asthma (n = 18)

§: p < 0.05 versus healthy subjects

K: p < 0.05 versus patients with MMA

### MUC1 mRNA expression was downregulated in the induced sputum cells of patients with asthma and correlated with asthma severity

**Fig. 1:**
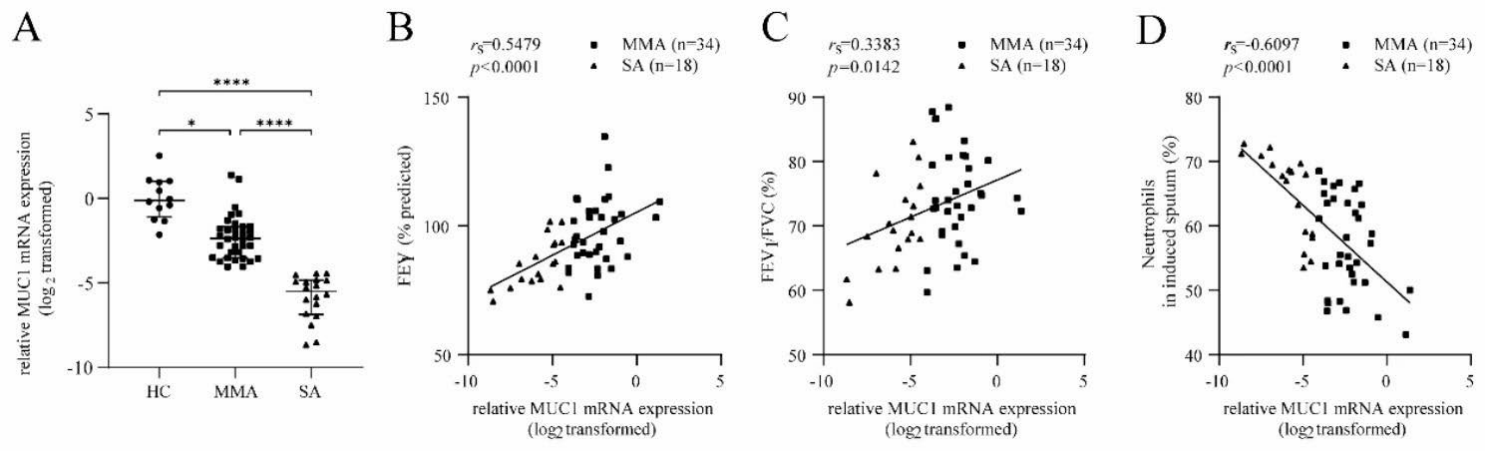
MUC1 mRNA expression was downregulated in the induced sputum of patients with asthma and correlated with asthma severity. Induced sputum cells from the HC (n = 12), MMA (n = 34), and SA (n = 18) groups were subjected to RT-qPCR to detect MUC1 expression in induced sputum cells. (**A**) The mRNA expression of MUC1 was detected via RT-qPCR. (**B–C**) The correlations of MUC1 mRNA expression in induced sputum cells with FEV_1_ (% predicted) and FEV_1_/FVC in asthma (n = 52). (**D**) The correlations of MUC1 mRNA expression with the proportion of neutrophils in induced sputum of patients with asthma (n = 52). HC = healthy controls (n = 12), MMA = mild-to-moderate asthma (n = 34), SA = severe asthma (n = 18). * *p* < 0.05; ** *p* < 0.01; *** *p* < 0.001; and **** *p* < 0.0001

RT-qPCR was performed to detect MUC1 expression in induced sputum cells. The mRNA expression of MUC1 was notably downregulated in induced sputum cells of patients with SA compared with those of patients with HC and MMA (Fig. 1A). The mRNA expression of MUC1 in induced sputum cells was negatively correlated with FEV1 (% predicted) and FEV_1_/FVC for patients with asthma (n = 52) (Fig. 1B and C) and positively correlated with the proportion of neutrophils in induced sputum (Fig. 1D). These results indicated that the mRNA expression of MUC1 was downregulated in the induced sputum cells of patients with asthma and negatively correlated with NA severity.

### mRNA expression of TLR4, MyD88, NLRP3, caspase-1, and IL-1β in induced sputum cells of patients with asthma was upregulated and related to the mRNA expression of MUC1

**Fig. 2:**
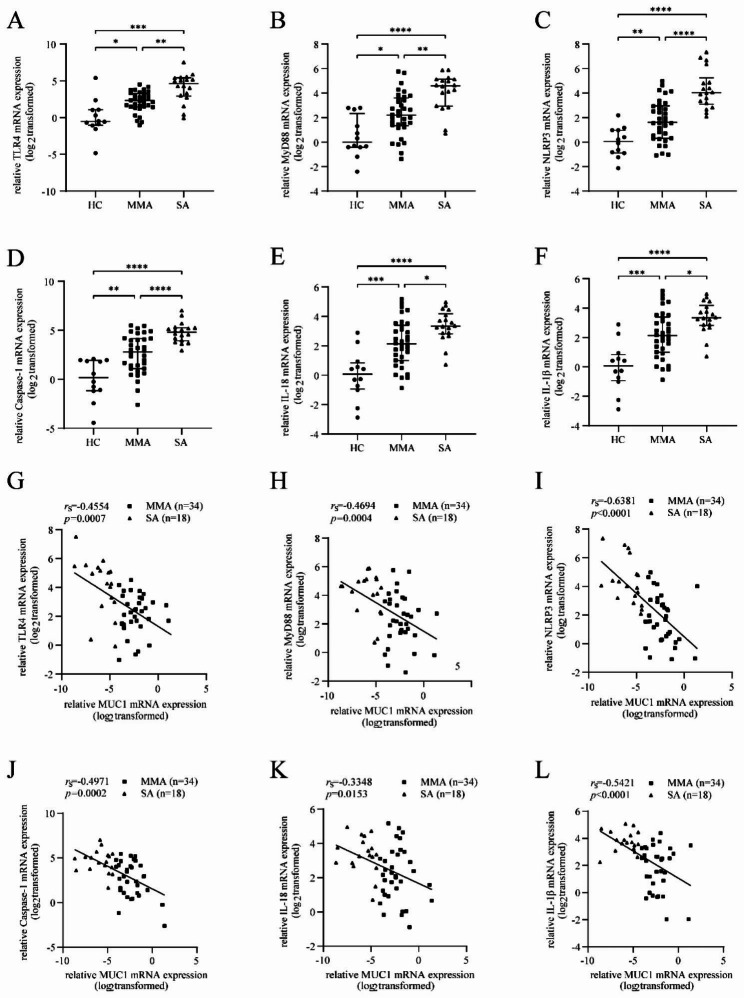
mRNA expression of TLR4, MyD88, and pyroptosis pathway molecules in the induced sputum of patients with asthma is upregulated and negatively related to the mRNA expression of MUC1. Induced sputum cells from the HC (n = 12), MMA (n = 34), and SA (n = 18) groups were subjected to RT-qPCR to detect the mRNA expression of TLR4, MyD88, NLRP3, caspase-1, IL-18, and IL-1β. (**A-F**) mRNA expression of TLR4, MyD88, NLRP3, caspase-1, IL-18, and IL-1β in induced sputum cells. (**G-L**) Correlation of the mRNA expression of MUC1 with the mRNA expression of TLR4, MyD88, NLRP3, caspase-1, and IL-1β in induced sputum cells of patients with asthma (n = 52). HC = healthy controls (n = 12), MMA = mild-to-moderate asthma (n = 34), SA = severe asthma (n = 18). * *p* < 0.05; ** *p* < 0.01; *** *p* < 0.001; and **** *p* < 0.0001

RT-qPCR was performed to detect the mRNA expression of TLR4, MyD88 and NLRP3, caspase-1, and IL-1β in induced sputum cells. The mRNA expression of TLR4, MyD88, NLRP3, caspase-1, IL-18, and IL-1β in induced sputum cells was significantly increased compared with that in patients with HC and MMA (Fig. 2A-I). Moreover, the mRNA expression of MUC1 was positively correlated with the mRNA expression of TLR4, MyD88, NLRP3, caspase-1 IL-18, and IL-1β (Fig. 2G-L).

### LPS upregulates MUC1 expression, activates the TLR4/MyD88/NF-κB pathway, and causes NLRP3 inflammasome-mediated pyroptosis and inflammation in vitro

**Fig. 3:**
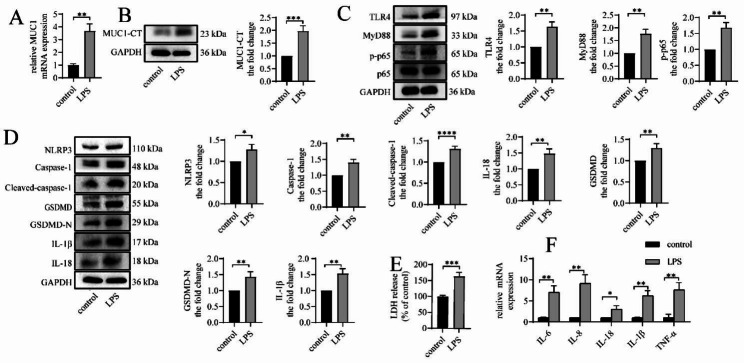
LPS upregulates MUC1 expression, activates the TLR4/MyD88/NF-κB pathway, and causes NLRP3 inflammasome-mediated pyroptosis and inflammation in vitro. BEAS-2B cells were stimulated with LPS (5 µg/mL) for 24 h. (**A**) The mRNA expression levels of MUC1 were detected via RT-qPCR. (**B–D**) The protein expression levels of MUC1, TLR4, MyD88, p-p65, p65, NLRP3, caspase-1, cleaved caspase-1, GSDMD, GSDMD-N, IL-18, and IL-1β were determined via immunoblotting. (**E**) LDH release into the culture medium was detected using LDH release assay. (**F**) The mRNA expression levels of IL-6, IL-8, IL-18, IL-1β, and TNF-α were detected using RT-qPCR. Data are expressed as mean ± SD (n = 3) and were analyzed using student t-test; *P < 0.05; **P < 0.01; ***P < 0.001; and ****p < 0.0001. LPS = lipopolysaccharide. LDH = lactate dehydrogenase

We stimulated BEAS-2B cells with LPS for 24 h in vitro to determine whether LPS affects the expression of MUC1 in BEAS-2B cells and found increased mRNA and protein expressions of MUC1 (Fig. 3A and B). Meanwhile, TLR4, MyD88 protein expression, and p65 phosphorylation level were upregulated in the LPS group compared with the control group (Fig. 3C). Furthermore, LPS increased the protein expression of NLRP3, caspase-1, cleaved caspase-1, GSDMD, GSDMD-N, IL-18, and IL-1β in cells (Fig. 3D) as well as increased the release of LDH (Fig. 3E), indicating that LPS induced pyroptosis in BEAS-2B cells. In addition, LPS increased the mRNA expression levels of the inflammatory factors IL-6, IL-8, IL-18, IL-1β, and TNF-α (Fig. 3F), triggering the inflammatory response of BEAS-2B cells.

### MUC1 downregulation aggravates LPS-induced TLR4/MyD88/NF-κB pathway activation and NLRP3 inflammasome-mediated pyroptosis and inflammation in vitro

**Fig. 4:**
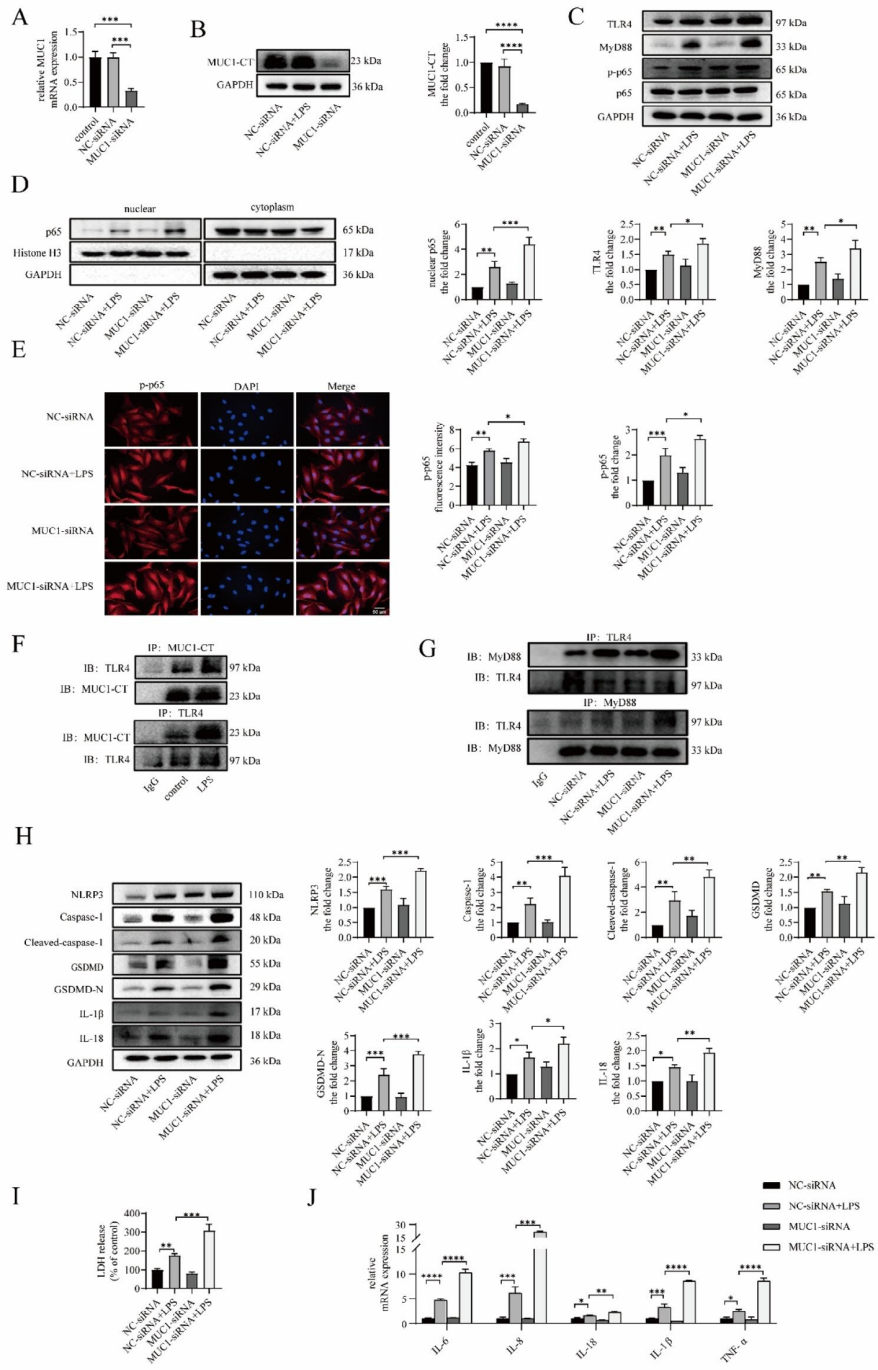
MUC1 downregulation aggravates LPS-induced TLR4/MyD88/NF-κB pathway activation and NLRP3 inflammasome-mediated pyroptosis and inflammation in vitro. BEAS-2B cells were treated with LPS (5 µg/mL) for 24 h after transfecting with MUC1-siRNA or negative control-siRNA for 24 h. (**A**) Protein interaction between MUC1 and TLR4 was detected using coimmunoprecipitation analyses. (**B**) The mRNA expression level of MUC1 was detected using RT-qPCR. (**C**) The protein expression levels of MUC1, TLR4, MyD88, p-p65, and p65 were determined via immunoblotting. (**D**) The levels of p65 proteins in the nuclear and cytoplasmic fractions were analyzed via immunoblotting. (**E**) The expression and localization of p-p65 were observed via immunofluorescence staining. Images were taken using a fluorescent microscope at 400x magnification. Red: p-p65; blue: DAPI; scale bar: 50 μm. (**F**) The protein interaction between MUC-CT and TLR4 was detected using COP-IP. (**G**) The protein interaction betweenTLR4 and MyD88 was detected using COP-IP. (**H**) The protein expression levels of NLRP3, caspase-1, cleaved caspase-1, GSDMD, GSDMD-N, IL-18, and IL-1β were determined via immunoblotting. (**I**) LDH release into the culture medium was detected via LDH release assay. (**J**) The mRNA expression levels of IL-6, IL-8, IL-18, IL-1β, and TNF-α were detected using RT-qPCR. Data are expressed as mean ± SD (n = 3) and were analyzed using one-way analysis of variance, *P < 0.05; **P < 0.01; ***P < 0.001; and ****p < 0.0001. LPS = lipopolysaccharide. LDH = lactate dehydrogenase

To demonstrate the effect of MUC1 on the TLR4/MyD88/NF-κB pathway and pyroptosis, BEAS-2B cells were transfected with MUC1-siRNA for 24 h before LPS intervention. Compared with the control and NC-siRNA groups, the mRNA and protein expression levels of MUC1 in the MUC1-siRNA group were significantly decreased (Fig. 4A and B). Compared with the NC-siRNA + LPS group, the protein expression levels of TLR4, MyD88, and p-p65 in the MUC1-siRNA + LPS group increased, and the p65 protein level in the nucleus also increased (Fig. 4C and D). Moreover, immunofluorescence staining of the cell slides revealed that the relative fluorescence intensity of p-p65 in the MUC1-siRNA + LPS group was increased (Fig. 4E). For pyroptosis, we found that LPS-induced NLRP3, caspase-1, cleaved caspase-1, GSDMD, GSDMD-N, IL-18, and IL-1β protein expression levels were further increased after MUC1 knockdown (Fig. 4H), and the LDH level in the cell supernatant was also further increased (Fig. 4I). At the same time, the mRNA expression levels of the inflammatory factors IL-6, IL-8, IL-18, IL-1β, and tumor necrosis factor (TNF)-α in BEAS-2B cells further increased after MUC1 knockdown (Fig. 4J). These results revealed that MUC1 knockdown facilitates TLR4/MyD88/NF-κB pathway activation, increases NLRP3-mediated pyroptosis, and aggravates inflammatory response, thereby indicating the protective role of MUC1 in asthma. Previous studies have revealed that MUC1 can competitively bind TLR5 and TLR3 with MyD88 through its cytoplasmic tail domain (MUC1-CT), thereby inhibiting NF-κB activation [29, 30]. Here, we used the CO-IP method to detect a protein interaction between MUC1-CT and TLR4 in BEAS-2B cells, and LPS enhanced their binding (Fig. 4F). Moreover, the binding of TLR4 and MyD88 significantly increased after MUC1-siRNA transfection, indicating that MUC1-CT can hinder the TLR4 and MyD88 binding (Fig. 4G).

### TAK-242 ameliorates TLR4/MyD88/NF-κB pathway activation and NLRP3 inflammasome-mediated pyroptosis and inflammation exacerbated by MUC1 downregulation in vitro

**Fig. 5:**
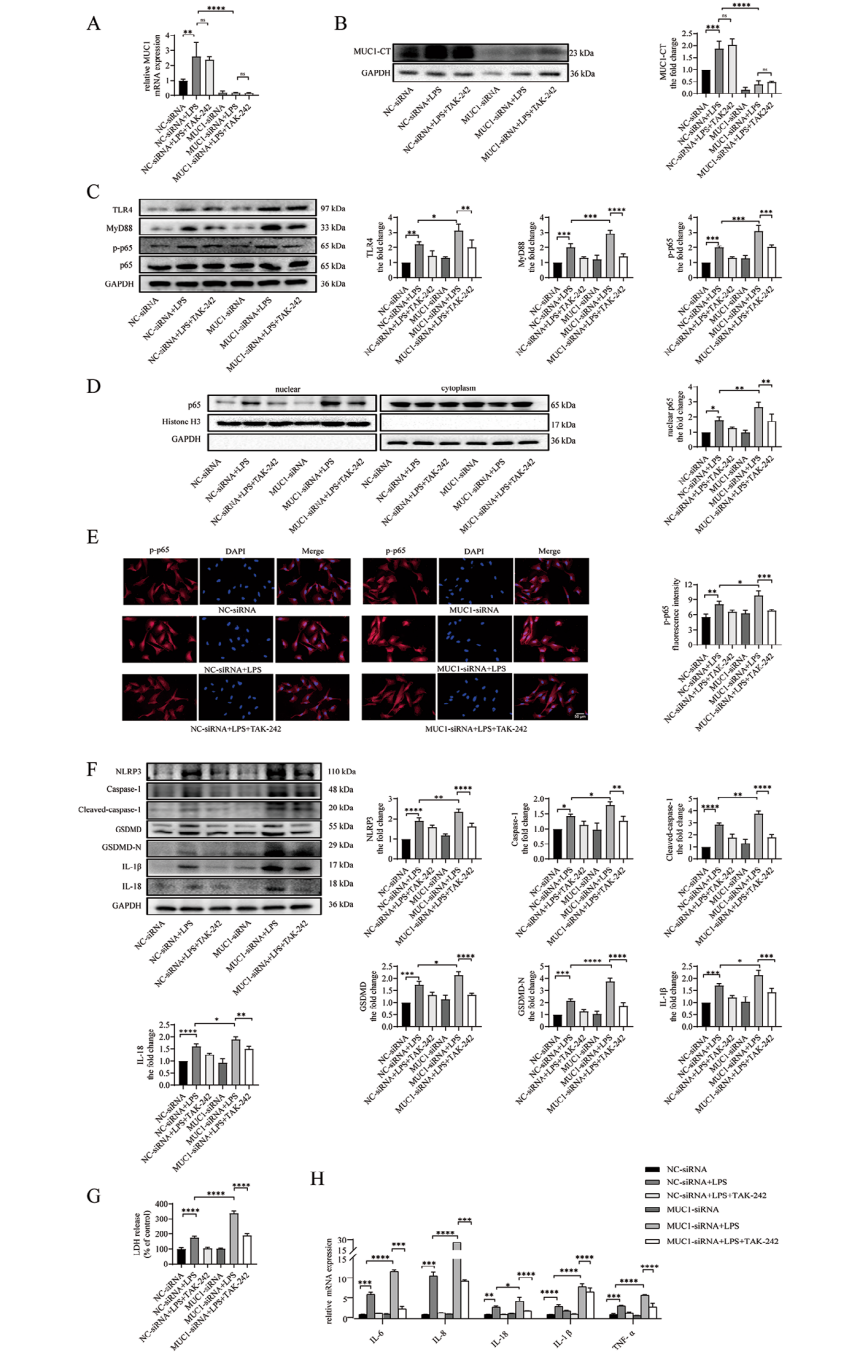
TAK-242 ameliorates TLR4/MyD88/NF-κB pathway activation and NLRP3 inflammasome-mediated pyroptosis and inflammation exacerbated by MUC1 downregulation in vitro. BEAS-2B cells were treated with TAK-242 (100 nM) for 2 h, followed by LPS (5 µg/mL) stimulating for 24 h, at 24 h after MUC1-siRNA or negative control-siRNA transfection. (**A**) The mRNA expression level of MUC1 was detected using RT-qPCR. (**B, C**) The protein expression levels of MUC1, TLR4, MyD88, p-p65, and p65 were determined via immunoblotting. (**D**) The p65 protein levels in the nuclear and cytoplasmic fractions were analyzed via immunoblotting. (**E**) The expression and localization of p-p65 were observed via immunofluorescence staining. Images were taken using a fluorescent microscope at 400x magnification. Red: p-p65; blue: DAPI; scale bar: 50 μm. (**F**) The protein expression levels of NLRP3, caspase-1, cleaved caspase-1, GSDMD, GSDMD-N, IL-18, and IL-1β were determined via immunoblotting. (**G**) LDH release into the culture medium was detected via LDH release assay. (**H**) The mRNA expression levels of IL-6, IL-8, IL-18, IL-1β, and TNF-α were detected using RT-qPCR. Data are expressed as mean ± SD (n = 3) and were analyzed using one-way analysis of variance, *p < 0.05; **p < 0.01; ***p < 0.001; and ****p < 0.0001. LPS = lipopolysaccharide; LDH = lactate dehydrogenase

Finally, we aimed to confirm the association between the inhibitory effect of MUC1 on pyroptosis and TLR4/MyD88/NF-κB pathway activation downregulation in vitro. We pretreated the cells with TAK-242, a specific TLR4 inhibitor, for 2 h before LPS stimulation. We found that TAK-242 pretreatment did not affect the expression level of MUCl (Fig. 5A and B). The expression of TLR4, MyD88, and p-p65 in the MUC1-siRNA + LPS + TAK-242 group was significantly reduced (Fig. 5C); moreover, nuclear p65 level (Fig. 5D) and the intensity of immunofluorescence for p-p65 (Fig. 5E) were reduced compared with the those in MUC1-siRNA + LPS group. Concurrently, the protein expression levels of NLRP3, caspase-1, cleaved caspase-1, GSDMD, GSDMD-N, IL-18, IL-1β, and LDH in the culture medium of the MUC1-siRNA + LPS + TAK-242 group were significantly lower than those of the MUC1-siRNA + LPS group (Fig. 5Fand G). The increased mRNA expression levels of the inflammatory factors IL-6, IL-8, IL-18, IL-1β, and TNF-α caused by MUC1 knockdown were partially reversed (Fig. 5H) after TAK-242 pretreatment. These results suggest that the protective effect of MUC1 against NLRP3-mediated pyroptosis and inflammation is related to its ability to block TLR4/MyD88/NF-κB pathway activation.

### TAK-242 partially ameliorates GO-203-exacerbated neutrophilic airway inflammation in OVA/LPS-induced asthmatic mouse

**Fig. 6:**
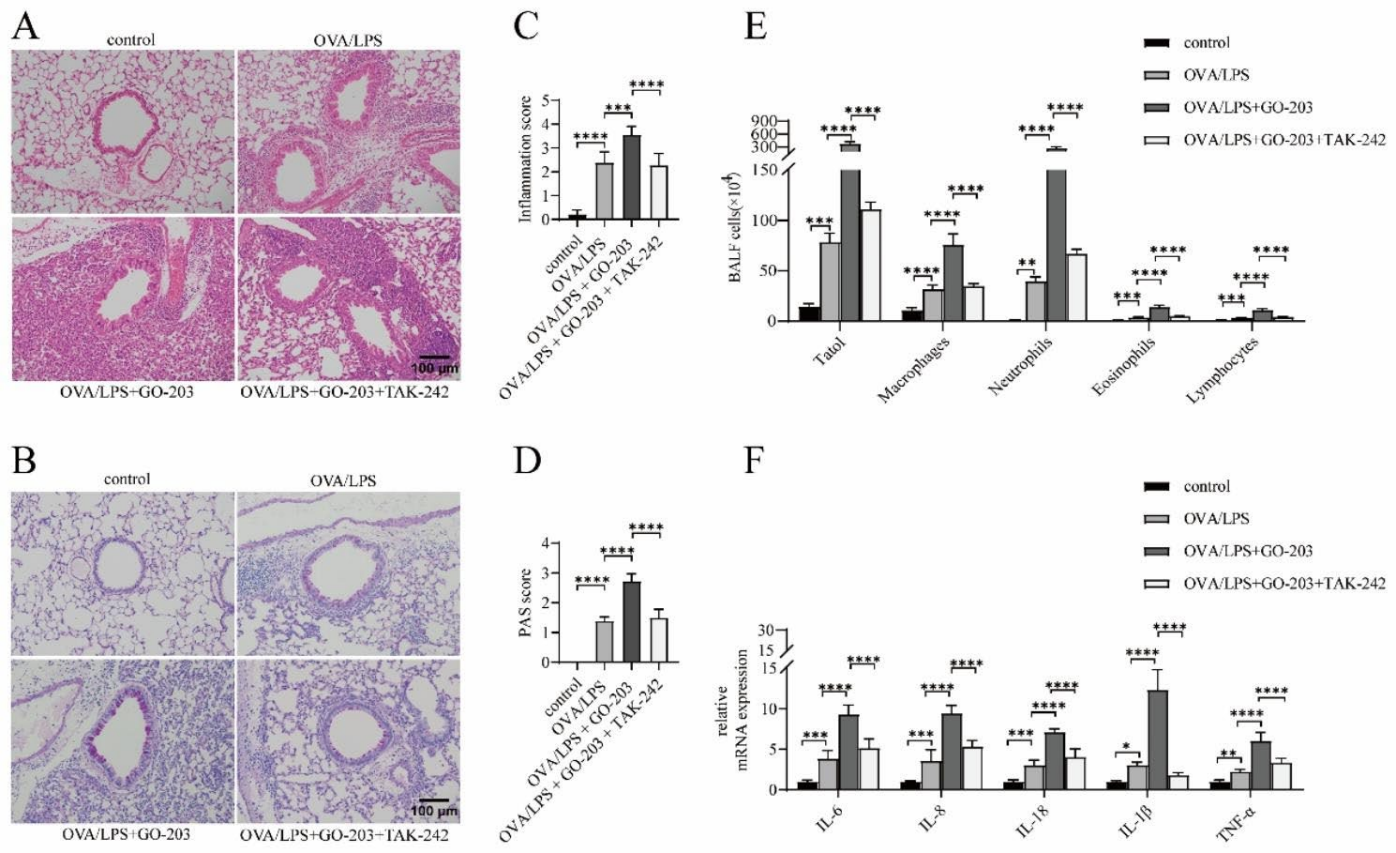
TAK-242 partially ameliorates GO-203-exacerbated neutrophilic airway inflammation in OVA/LPS-induced asthmatic mouse. (**A, B**) HE staining and PAS staining were performed on lung sections in the indicated groups. Images were taken under a microscope at 200× magnification. Scale bar: 50 μm. (**C, D**) Inflammatory infiltration and hyperplasia of goblet cells were quantified using inflammation and PAS scores. (**E**) Statistical analysis of the total inflammatory cells, macrophages, neutrophils, eosinophils, and lymphocytes in BALF. (**F**) The mRNA expression levels of IL-6, IL-8, IL-18, IL-1β, and TNF-α in lung tissues were detected using RT-qPCR. LDH = lactate dehydrogenase. Data are expressed as mean ± SD (n = 6 animals per group) and were analyzed using one-way analysis of variance,*p < 0.05; **p < 0.01; ***p < 0.001; and ****p < 0.0001

Previous studies have revealed that the anti-inflammatory activity of MUC1 in TLR pathway activation mainly depends on its intracellular cytoplasmic tail (MUC1-CT) inhibition [27, 31]. GO-203, an analog of MUC1-CT, can penetrate the cell membrane and bind to the CQC motif of MUC-CT, thereby blocking its intracellular signal transduction function [32] and the TLR pathway regulation function [26, 33]. We induced C57BL/6J mice with OVA/LPS to establish **a**sthmatic models and used GO-203 and TAK-242 to block the MUC1 and TLR4 functions, respectively, to verify the results obtained in cell experiments in vivo. The results were consistent with what we expected. The inflammation around the airway, goblet cell hyperplasia, inflammation score, and PAS score in the OVA/LPS + GO-203 group (Fig. 6A and B) were significantly increased compared with the OVA/LPS group (Fig. 6C and D). The total number and differential counts of inflammatory cells in the BALF significantly increased, with the most significant increase observed in neutrophils, in the BALF of mice in the OVA/LPS + GO-203 group (Fig. 6E). Concurrently, the mRNA expressions of the inflammatory factors IL-6, IL-8, IL-18, IL-1β, and TNF-α in the lung tissue of mice in the OVA/LPS + GO-203 group were significantly increased (Fig. 6F). These results indicated that GO-203 blocked the function of MUC1-CT to aggravate neutrophil inflammation in the airways of OVA/LPS-induced asthmatic mouse, suggesting the protective effect of MUC1 on neutrophilic airway inflammation in patients with asthma. Comparison of the OVA/LPS + GO-203 and OVA/LPS + GO-203 + TAK-242 groups revealed that the abovementioned airway inflammation indicators were significantly suppressed in the OVA/LPS + GO-203 + TAK-242 group. Furthermore, we observed that TAK-242 partially reversed the increased neutrophilic airway inflammation caused by MUC1 function inhibition in OVA/LPS-induced asthmatic mouse.

### TAK-242 partially downregulates GO-203-caused TLR4/MyD88/NF-κB pathway activation and NLRP3 inflammasome-mediated pyroptosis in OVA/LPS-induced asthmatic mouse

**Fig. 7:**
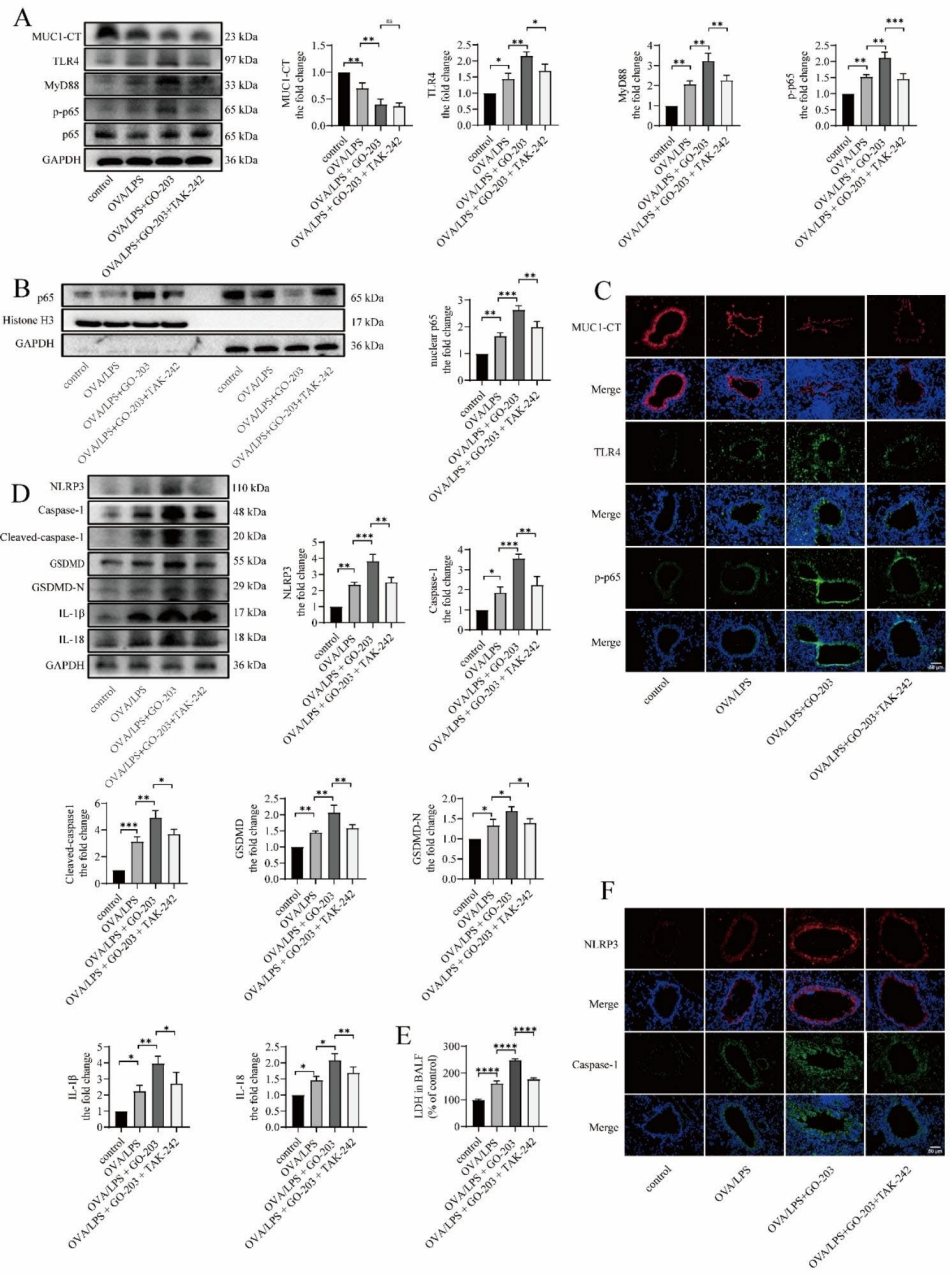
TAK-242 downregulates GO-203-caused TLR4/MyD88/NF-κB pathway activation and NLRP3 inflammasome-mediated pyroptosis in OVA/LPS-induced asthmatic mouse. (**A**) The protein expression levels of MUC1, TLR4, MyD88, p-p65, and p65 in lung tissue were determined via immunoblotting. (**B**) The expression of MUC1, TLR4, and p-p65 in the bronchi were determined via immunofluorescence staining. Images were taken using a fluorescent microscope at 400× magnification. Red: MUC1; green: TLR4 and p-p65; blue: DAPI; scale bar: 50 μm. (**C**) The p65 protein levels in the nuclear and cytoplasmic fractions in lung tissue were analyzed via immunoblotting. (**D**) The protein expression levels of NLRP3, caspase-1, cleaved caspase-1, GSDMD, GSDMD-N, IL-18, and IL-1β in lung tissue were determined via immunoblotting. (**E**) LDH release into BALF was detected via LDH release assay. (**F**) The expression of NLRP3 and caspase-1 in the bronchi were determined via immunofluorescence staining. Images were taken using a fluorescent microscope at 400× magnification. Red: NLRP3; green: caspase-1; blue: DAPI; scale bar: 50 μm. LDH = lactate dehydrogenase. Data are expressed as mean ± SD (n = 6 animals per group) and were analyzed using one-way analysis of variance, *p < 0.05; **p < 0.01; ***p < 0.001; and ****p < 0.0001

We found that the expression level of MUC1 was decreased in the OVA/LPS group compared with the control group (Fig. 7A and B), whereas the expression levels of TLR4, MyD88, p-p65, and nuclear p65 (Fig. 7A-C) were significantly increased, as detected via western blotting and immunofluorescence staining. In addition, the protein expression levels of NLRP3, caspase-1, cleaved caspase-1, GSDMD, GSDMD-N, IL-18, and IL-1β (Fig. 7D and E) were increased, indicating that MUC1, TLR4/MyD88/NF-κB pathway, and NLRP3-induced pyroptosis are involved in the pathogenesis of asthma. The protein expression levels of TLR4, MyD88, and p-p65 in the lung tissue as well as nuclear p65 level of mice in the OVA/LPS + GO-203 group were significantly increased compared with the OVA/LPS group (Fig. 7A-C). Moreover, the LDH level in the OVA/LPS + GO-203 group was significantly higher than that in the OVA/LPS group (Fig. 7E), and the protein expression levels of NLRP3, caspase-1, cleaved caspase-1, GSDMD, GSDMD-N, IL-18, and IL-1β were significantly increased (Fig. 7D and E). These results indicated that significant inhibition of the signal transduction function of MUC1 facilitates TLR4/MyD88/p-p65 pathway activation and NLRP3-mediated pyroptosis. Furthermore, TLR4/MyD88/NF-κB pathway activation and NLRP3-mediated pyroptosis were simultaneously inhibited in the OVA/LPS + GO-203 group compared with the OVA/LPS + GO-203 group. Overall, TAK-242 downregulates GO-203-caused TLR4/MyD88/NF-κB pathway activation and NLRP3 inflammasome-mediated pyroptosis in OVA/LPS-induced asthmatic mouse.

## Discussion

Neutrophilic airway inflammation is considered an important issue in asthma management and is often accompanied by corticosteroid resistance, progressive lung function decline, and frequent asthma exacerbations [34]. At present, treatment methods for asthma mainly target eosinophilic inflammation, and the symptom control of patients with asthma having neutrophilic airway inflammation is often not satisfactory. Therefore, the underlying mechanism and alternative therapeutic targets for neutrophilic airway inflammation in asthma should be explored.

MUC1 has been considered to have a potential value in the treatment of SA because of its role in regulating the sensitivity of glucocorticoids [15]. However, neutrophilic airway inflammation regulation by MUC1 is unclear in asthma. The present study revealed that MUC1 expression in induced sputum of patients with asthma was downregulated and was related to asthma severity. This is consistent with the results of a previous study on human bronchial epithelial cells and blood neutrophils [15]. Moreover, we discovered that MUC1 expression was significantly negatively correlated with the proportion of neutrophils in induced sputum of patients with asthma, indicating that MUC1 may have an inhibitory effect on neutrophil airway inflammation in asthma. We did not perform bronchial biopsy and collect airway epithelial cells from the participants, which may represent a limitation of this study, considering ethics and patient acceptance. However, a previous study [15] reported changes in the expression levels of MUC1 in bronchial epithelial cells and blood neutrophils of patients with asthma, and our study supplemented the changes in induced sputum cells. Interestingly, Anil KM et al. [35] found that MUC1 expression was upregulated in most patients with asthma who were sensitive and exposed to a pigeon allergen. Their results are inconsistent with our results, possibly due to the following reasons: First, the samples they collected and the MUC1 structural fragments they detected were different from those in our study. They detected the protein expression of the circulating form of MUC1, a sialylated sugar chain of the MUC1 ectodomain, in the serum, whereas we detected MUC1 gene expression in induced sputum cells. Second, their study did not include a healthy control group for comparison with patients with asthma. Moreover, they did they group and compare patients according to the severity of asthma, as performed in our study. In addition, they compared patients with asthma with positive and negative pigeon allergens and found that MUC1 expression in the serum of patients with positive pigeon allergens was higher than that in the serum of patients with negative pigeon allergens, suggesting that the circulating form of MUC1 is a biomarker in pigeon-sensitive asthma patients [35]. However, the mechanism by which the circulating form of MUC1 is upregulated in patients with positive pigeon allergens remains unknown and is worth exploring in the future.

NLRP3 inflammasome-mediated pyroptosis has been thought to be an important trigger of neutrophil airway inflammation in asthma [36–38]. Previous studies have reported that TLR4 can promote NF-κB activation by interacting with the adapter molecule MyD88, induce NLRP3 inflammasome and caspase-1 activation, and trigger pyroptosis [39–41]. Concurrently, the negative regulatory effect of MUC1 on the TLR4/MyD88/NF-κB pathway has been described in other diseases, such as acute lung injury and acute kidney injury [26, 42]. However, whether MUC1 regulates NLRP3 inflammasome-mediated pyroptosis through the TLR4/MyD88/NF-κB pathway in asthma remains unknown. We examined the expression levels of TLR4/MyD88/NF-κB pathway- and NLRP3 inflammasome-mediated pyroptosis-related molecules in induced sputum cells. The results revealed that the expression of these molecules in induced sputum cells of patients with asthma was significantly increased compared with that in healthy subjects. Furthermore, consistent with our hypothesis, the expression of these molecules was significantly negatively correlated with the expression of MUC1.

We discovered that NLRP3-mediated pyroptosis was significantly increased after the reduction of MUC1 expression in in vitro experiments through stimulation of BEAS-2B with LPS and successive transfecton with MUC1-siRNA; this revealed the potential of MUC1 in reducing neutrophilic inflammation in asthma that was closely related to NLRP3 inflammasome-mediated-pyroptosis. Previous studies have revealed that MUC1 can inhibit NLRP3 inflammasome activation in *Helicobacter pylori* gastritis and bacterial infection, but they mainly studied NLRP3 inflammasome as an inflammatory mediator [43, 44]. In contrast, this study regarded NLRP3 inflammasome activation as an initiation of pyroptosis and revealed the downregulation effect of MUC1 on pyroptosis in asthma. We further revealed that TLR4 inhibition attenuated the upregulation of pyroptosis induced by MUC1 knockdown using TAK-242. These results demonstrated that the TLR4/MyD88/NF-κB pathway was a bridge between MUC1 and NLRP3 inflammasome-mediated pyroptosis. Furthermore, this study adds to the evidence that MUC1 regulates the TLR4/MyD88/NF-κB pathway and NLRP3 inflammasome-mediated pyroptosis in patients with asthma.

However, the exact mechanism of how MUC1 regulates the TLR4/MyD88/NF-κB pathway is unclear. Protein interactions were reported between MUC1 and some TLRs, including TLR5, TLR3, and TLR9 [29, 31, 45]. Gibier, Jean-Baptiste et al. [26] demonstrated the interaction between MUC1 and TLR4 in HEK-293 cells using proximity ligation assay and revealed that MUC1 can interact with TLR4 through its intracellular CT domain, act as a competitive inhibitor of MyD88, and reduce NF-κB activation. Therefore, we also detected the interaction between MUC1-CT and TLR4 using CO-IP in BEAS-2B cells and found that MyD88 recruited by TLR4 was significantly increased in BEAS-2B cells stimulated with LPS after MUC1 knockdown. These results indicate that the interaction between MUC1-CT and TLR4 is one of the mechanisms for MUC1 to regulate the TLR4/MyD88/NF-κB pathway. Moreover, the expression of TLR4 was significantly upregulated after transfection with MUC1-siRNA, consistent with other studies [26, 33]. Yu-Ming Wang recently reported that MUC1 inhibited the expression of TLR4 by stabilizing the transcription factor HIF-α [33]. We did not verify this nor did we deeply explore other possible mechanisms by which MUC1 affects the expression level of TLR4; this can be a topic for future research.

GO-203, an analog of MUC1-CT, can bind to the CQC motif of MUC-CT, thereby blocking its intracellular signal transduction function [32] and the regulation function of the TLR pathway [26, 33]. Finally, intraperitoneal injection with GO-203 in OVA/LPS-induced asthmatic mice aggravated neutrophil inflammation in their airways and increased TLR4/MyD88/NF-κB pathway activation and NLPP3 inflammasome-mediated pyroptosis. Moreover, TAK-242, a specific TLR4 inhibitor, partially reversed this change. Consistent with the in vitro results, these in vivo results more intuitively clarified the role of MUC1 in reducing neutrophilic airway inflammation in asthma. However, economic conditions to construct a MUC1 knockout mouse model were insufficient, being a limitation of this study. Fortunately, many studies have recognized the inhibitory effect of GO-203 on MUC1-CT in the lung [26, 46].

This study explored the inhibitory effect of MUC1 on NLRP3 inflammasome-mediated pyroptosis in patients with asthma and revealed that one of the underlying mechanisms is the downregulation of TLR4/MyD88/NF-κB pathway activation. Several previous studies have suggested that neutrophilic airway inflammation in asthma may be effectively reduced by blocking NLRP3 inflammasome-mediated pyroptosis [10, 47, 48]; however, the results of clinical trials have been disappointing. For example, clinical trials of the NLRP3 inhibitor MCC950 were halted due to its toxicity [49]. Canakinumab, an IL-1β monoclonal antibody, is no longer being studied as a therapeutic drug for asthma because of its complicated mechanism and unsatisfactory effect [50]. The role of some other pyroptosis inhibitors in asthma remains unclear. Therefore, the results of this study are clinically significant. We explored a long signaling pathway involved in neutrophil airway inflammation in asthma, which offers many alternative therapeutic targets for neutrophilic asthma. Targeting the upstream of NLRP3 inflammasome-mediated pyroptosis and simultaneously inhibiting multiple targets may have a better therapeutic effect on neutrophil airway inflammation in asthma.

The greatest significance of this study is to reveal the potential protective role of MUC1 in neutrophil airway inflammation in patients with asthma. In general, neutrophilic airway inflammation and glucocorticoid resistance are important factors for poor asthma control, and an inherent correlation exists between the two [51]. Previous studies have demonstrated that MUC1 deficiency mediates corticosteroid insensitivity in patients with asthma [15, 18]. This study shows the role of MUC1 in attenuating neutrophil-induced airway inflammation, further emphasizing the importance of MUC1 in the pathogenesis of asthma, which may have a potential value for developing new treatment strategies for refractory asthma. However, the reason for MUC1 expression downregulation in the course of asthma remains unknown, and more research may be needed in the future to prevent MUC1 deficiency or increase MUC1 expression in asthma.

**Fig. 8:**
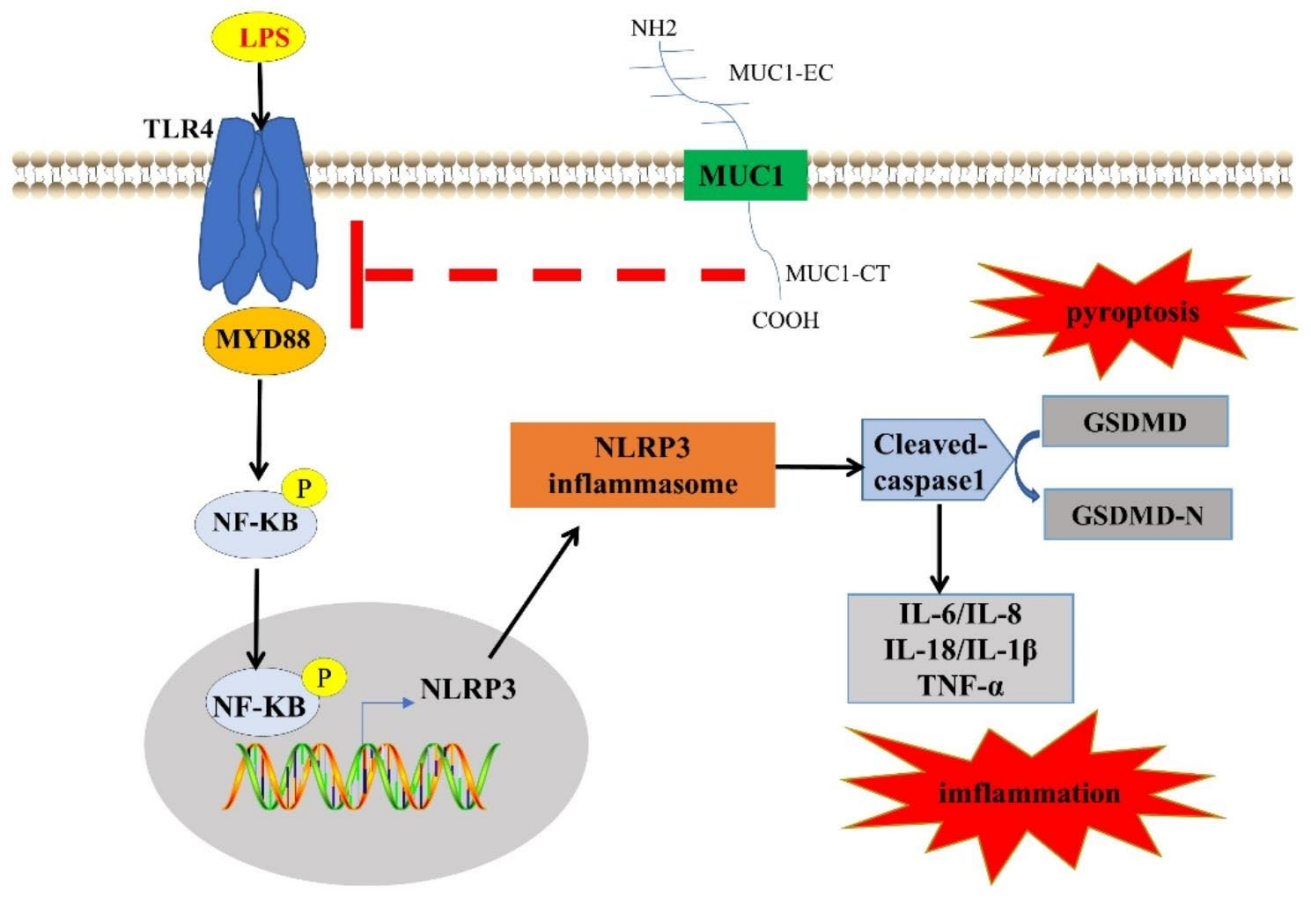
Mechanisms underlying MUC1 regulation of neutrophilic airway inflammation in asthma. MUC1-CT can interact with TLR4 and hinder the interaction between TLR4 and MyD88, thereby reducing the phosphorylation and nuclear translocation of NF-κB and reducing NLRP3 inflammasome-mediated pyroptosis and neutrophilic airway inflammation. Therefore, MUC1 downregulation in the airways of patients with asthma may be a mechanism of neutrophilic airway inflammation

## Conclusion

In summary, this study revealed that MUC1 was downregulated in induced sputum of patients with asthma and played an important role in neutrophilic airway inflammation. Mechanistic studies revealed that MUC1 reduced NLRP3 inflammasome-mediated pyroptosis by inhibiting TLR4/MyD88/NF-κB pathway activation, thereby suppressing neutrophilic airway inflammation in patients with asthma. This study suggested a possible mechanism of neutrophilic airway inflammation in patients with asthma and emphasized the anti-inflammatory function of MUC1 in neutrophilic asthma, thereby indicating that MUC1 is of a potential value to develop new asthma treatment strategies.

### Electronic supplementary material

Supplementary tables

## Data Availability

The datasets supporting the conclusions of this article are included within the article and its additional files. Any other data and materials generated in this study are available from the corresponding author upon request.

## Abbreviations

GRα: Glucocorticoid receptor alpha
H&E: Hematoxylin and eosin
HC: Healthy controls
LPS: Lipopolysaccharide
MMA: Mild-to-moderate asthma
MUC1-CT: Mucin 1 cytoplasmic tail
NLRP3: Nucleotide-binding oligomerization domain-like protein3
PAS: Periodic acid–Schiff stain
PBS: Phosphate-buffered saline
SA: Severe asthma
SD: Standard deviation
TLR: Toll-like receptor
