# Supplementary material for: MUC1 attenuates neutrophilic airway inflammation in asthma by reducing NLRP3 inflammasome-mediated pyroptosis through the inhibition of the TLR4/MyD88/NF-κB pathway
Source: Respir Res. 2023 Oct 25;24:255. doi: 10.1186/s12931-023-02550-y (PMC10601133; doi:10.1186/s12931-023-02550-y)
Supplement: Supplementary file 1 — Supplementary tables [file 12931_2023_2550_MOESM1_ESM.doc]

Supplementary tables

Table S1. Primer sequences

| Primers | Forward | Reverse |
| --- | --- | --- |
| β-actin(human)  (for induced sputum) | 5′-CATGTACGTTGCTATCCAGGC-3′ | 5′-CTCCTTAATGTCACGCACGAT-3′ |
| GAPDH (human)  (for BEAS-2B) | 5′-GGAGCGAGATCCCTCCAAAAT-3′ | 5′-GGCTGTTGTCATACTTCTCATGG-3′ |
| MUC1 (human) | 5′-TGCCGCCGAAAGAACTACG-3′ | 5′-TGGGGTACTCGCTCATAGGAT-3′ |
| IL-6 (human) | 5′-ACTCACCTCTTCAGAACGAATTG-3′ | 5′-CCATCTTTGGAAGGTTCAGGTTG-3′ |
| IL-8 (human) | 5′-ACTGAGAGTGATTGAGAGTGGAC-3′ | 5′-AACCCTCTGCACCCAGTTTTC-3′ |
| IL-18 (human) | 5′-ACTGAGAGTGATTGAGAGTGGAC-3′ | 5′-AACCCTCTGCACCCAGTTTTC-3′ |
| IL-1β (human) | 5′-ATGATGGCTTATTACAGTGGCAA-3′ | 5′-GTCGGAGATTCGTAGCTGGA-3′ |
| TNF-α (human) | 5′-GCCTCTTCTCCTTCCTGATCG-3′ | 5′-TCGAGAAGATGATCTGACTGCC-3′ |
| NLRP3(human) | 5′-CCCGACAGTGGATATAGAACAGA-3′ | 5′-CGTGAGTCCCATTAAGATGGAGT-3′ |
| Caspase-1(human) | 5′-TTTCCGCAAGGTTCGATTTTCA-3′ | 5′-GGCATCTGCGCTCTACCATC-3′ |
| TLR4 (human) | 5′-AGACCTGTCCCTGAACCCTAT-3′ | 5′-CGATGGACTTCTAAACCAGCCA-3′ |
| MyD88 (human) | 5′-GGCTGCTCTCAACATGCGA-3′ | 5′-CTGTGTCCGCACGTTCAAGA-3′ |
| GADPH (mouse) | 5′-AGGTCGGTGTGAACGGATTTG-3′ | 5′-TGTAGACCATGTAGTTGAGGTCA-3′ |
| IL-6 (mouse) | 5′-TAGTCCTTCCTACCCCAATTTCC-3′ | 5′-TTGGTCCTTAGCCACTCCTTC-3′ |
| IL-8 (mouse) | 5′-TGTTGAGCATGAAAAGCCTCTAT-3′ | 5′-AGGTCTCCCGAATTGGAAAGG-3′ |
| IL-18 (mouse) | 5′-GTGAACCCCAGACCAGACTG-3′ | 5′-CCTGGAACACGTTTCTGAAAGA-3′ |
| IL-1β (mouse) | 5′-GAAATGCCACCTTTTGACAGTG-3′ | 5′-TGGATGCTCTCATCAGGACAG-3′ |
| TNF-α (mouse) | 5′-AATGGCCTCCCTCTCATCAGT T-3′ | 5′-CCACTTGGTGGTTTGCTACGA-3′ |

Table S2. Primary antibody

| Antibody | Antibody manufacturers | Antibody catalog number |
| --- | --- | --- |
| GAPDH | Proteintech | 60004-1-Ig |
| MUC1-CT | Abmart | P15941 |
| TLR4 | Abmart | TA7017 |
| MyD88 | Abmart | TA5195 |
| p65 | Abmart | T55034 |
| p-p65 | Abmart | TA2006 |
| NLRP3 | ABclonal | A5652 |
| Caspase-1 | ABclonal | A16792 |
| GSDMD | ABclonal | A20197 |
| IL-18 | Proteintech | 10663-1-AP |
| IL-1β | ABclonal | A19635 |
